# Pathogen resistance may be the principal evolutionary advantage provided by the microbiome

**DOI:** 10.1098/rstb.2019.0592

**Published:** 2020-08-10

**Authors:** Michael R. McLaren, Benjamin J. Callahan

**Affiliations:** 1 Department of Population Health and Pathobiology, North Carolina State University, Raleigh, NC 27607, USA; 2 Bioinformatics Research Center, North Carolina State University, Raleigh, NC 27695, USA

**Keywords:** colonization resistance, commensal bacteria, defensive symbionts, evolution of immunity, host–microbiome interactions, microbiome

## Abstract

To survive, plants and animals must continually defend against pathogenic microbes that would invade and disrupt their tissues. Yet they do not attempt to extirpate all microbes. Instead, they tolerate and even encourage the growth of commensal microbes, which compete with pathogens for resources and via direct inhibition. We argue that hosts have evolved to cooperate with commensals in order to enhance the pathogen resistance this competition provides. We briefly describe competition between commensals and pathogens within the host, consider how natural selection might favour hosts that tilt this competition in favour of commensals, and describe examples of extant host traits that may serve this purpose. Finally, we consider ways that this cooperative immunity may have facilitated the adaptive evolution of non-pathogen-related host traits. On the basis of these observations, we argue that pathogen resistance vies with other commensal-provided benefits for being the principal evolutionary advantage provided by the microbiome to host lineages across the tree of life.

This article is part of the theme issue ‘The role of the microbiome in host evolution’.

## 1. Introduction

Multicellular life evolved and is continually reborn into a world of microbes. To these diverse microbial creatures, multicellular hosts are another potential source of food and shelter to compete over. To the host these microbes range from harmless features of the environment, to new tools for extracting resources, to bringers of destruction. A typical microbiome is largely composed of commensal microbes that grow and persist on hosts in a relatively benign fashion. For most of these commensals, a plant or animal host is simply a hospitable growing environment, protected from certain environmental stresses and containing high nutrient concentrations near their surface owing to nutrient acquisition and excretion activities. However, nutrient concentrations are higher still within the host’s tissues, making the host effectively a weakly protected nutrient cache [1]. As a result, other microbial taxa have evolved pathogenic lifestyles based on breaking into these caches, rapidly consuming what they can, and moving on to the next host. A single human may encounter thousands of pathogenic microbes in its lifetime [2], and before the rise of modern medicine pathogens caused roughly half of human deaths [3]. Although such estimates are lacking for other species, there is no doubt that pathogens ultimately cause a large fraction of mortality and lost fecundity in wild plants and animals. To survive, multicellular life has evolved a vast array of costly defence mechanisms. But despite the dangers, it cannot simply shut itself off from the microbial world.

Plant and animal hosts face competing constraints of needing to constantly exchange nutrients and waste with the environment and needing protection from the microbes in the environment that would exploit them. A host can physically contain microbes to some extent, but cannot completely isolate itself from the environment and still grow and survive. Hosts must have semi-porous surfaces and openings where matter and microbes can enter. Host defence mechanisms such as production of antimicrobial compounds, programmed death in infected cells, and activation of innate and adaptive immune responses are effective deterrents against many pathogens, but bear heavy costs in the form of self-inflicted tissue damage and diversion of resources from normal homeostasis and growth processes [4–7].

Meanwhile the microbes within a host constrain each other through competition. The many microbial species within a host compete with each other for space and nutrients, directly inhibit each other through the secretion of toxic chemicals into the environment or directly into competing cells, and indirectly inhibit each other through their shared viral predators and the immune systems of their hosts [8–11]. To colonize a host with an established microbiome, new entrants must survive this fierce competition from the existing community [12–14].

**Figure 1.**
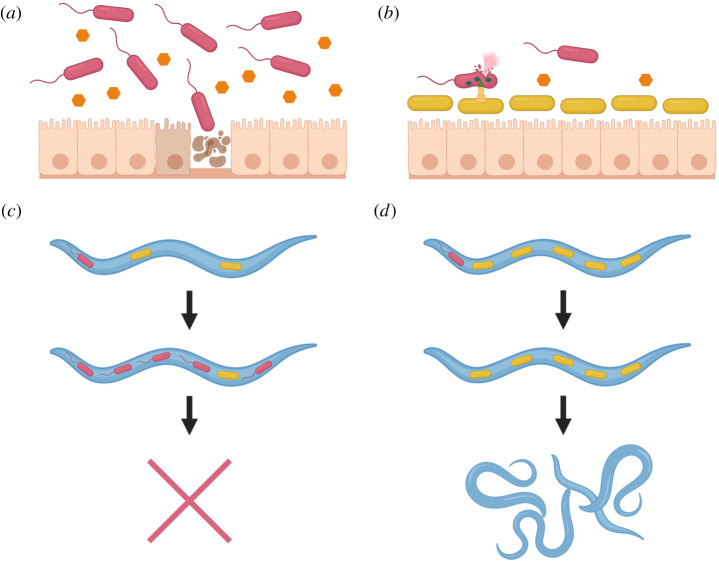
Competition between commensal and pathogenic microbes affects the survival and evolution of their hosts. (*a,b*) All plants and animals encounter pathogenic microbes (pictured here as red flagellated bacilli), which seek to damage the host’s epithelial and other protective tissues in order to extract the nutrients within, and commensal microbes (yellow non-flagellated bacilli), which can live on hosts while causing minimal damage. Commensals restrict the nutrients (orange hexagons) and physical access to vulnerable host tissues available to pathogens and also directly harm pathogens with secretion systems and other microbial weapons. (*c,d*) Under strong pathogen pressure, hosts that acquire and maintain commensal microbiomes that are more effective at resisting a broad range of pathogens will tend to survive and leave more offspring than their competitors.

From the confluence of constraints on hosts and microbes emerges a potentially profitable strategy for the host: rather than attempting to shut out all microbes, leverage the presence of commensal microbes for pathogen defence. For a pathogen invading a host microbiome, competition from commensals is expected to reduce the pathogen’s ability to survive and grow within the community and restrict physical access to vulnerable host tissues (figure 1*a*,*b*). Thus, hosts gain pathogen resistance from commensals simply as a by-product of microbial competition. Host traits that differentially favour the growth of commensals over pathogens can further increase the fraction of invading pathogens that will be outcompeted by the microbiome’s residents. By reducing the rate of new infections, these traits can confer strong fitness benefits to the host (figure 1*c*,*d*). Such traits may be more evolutionarily accessible and/or less costly than suppressing microbial colonization entirely, and could reduce the resources expended and costs accrued by intense host-based pathogen defence. In the remainder of this article, we explore theoretical arguments and empirical evidence that hosts have evolved to make commensals a key part of pathogen defence. We also consider ways that pathogen resistance provided by commensals may have facilitated the adaptive evolution of other (non-pathogen-related) host traits. On the basis of these observations, we argue that the pathogen resistance vies with other commensal-provided benefits for being the principal evolutionary advantage provided by the microbiome to host lineages across the tree of life.

## 2. Pathogens compete with commensals to inhabit the host

All microbes require nutrients and a hospitable space to survive and reproduce. The relative abilities of various species to compete for these limited resources determines which ones are able to colonize and persist in an ecosystem. This competition has led to the evolution of a vast array of competitive phenotypes among microbes, i.e. adaptations that enhance a microbe’s ability to outcompete others. The outcome of competition within a host is also influenced by factors external to the microbes (though often under host control), such as resource availability and the presence of enemies such as predators and the host immune system. Here we briefly review how the competition between commensals and pathogens helps determine the outcome of a pathogen encounter with a host and its microbiome.

*Exploitative competition* refers to the indirect negative impact species have on themselves and each other through the consumption of commonly used resources [9,15]. Pathogens and commensals both require carbohydrates, minerals and other nutrients to grow. In many examples across plants and animals, consumption of these nutrients by commensals has been found to limit the growth of pathogens [13,16–23]. Commensals adhere and grow to cover host surfaces, limiting space for pathogens to find purchase [13,24]. Many adaptations to enhance a species’ resource-exploitation ability are available to both commensals and pathogens, such as the formation of biofilms to secure space [13,24] and the secretion of siderophores to scavenge iron [13,25,26]. In addition, pathogens have evolved methods to access resources that are unavailable to commensals as they involve directly harming the host. For example, many plant and animal pathogens carry enzymes to degrade host cell walls [27,28], and the gut pathogen *Salmonella* Typhimurium uses molecules produced by a deliberately triggered inflammatory response for its own respiration [29].

Exploitative competition from commensals can help determine whether a pathogen successfully infects the host. A pathogen infection in an established microbiome resembles the growth of an invasive species as traditionally studied by ecologists [30]. As such, we can use community ecology theory as applied to biological invasions to consider the outcome of a pathogen encounter [31]. A single commensal species will tend to increase in population density until it has decreased resources to the point where it is just able to maintain its current density. Pathogens that are better able to exploit the available resources than the commensal, by maintaining positive growth where the commensal cannot, can invade the community, albeit with a reduced growth rate relative to in an uninhabited community. Other pathogens will be unable to have positive growth on the remaining resources and thus be effectively resisted. A diverse community can more completely use the resources in a given environment and thus may resist more invaders [31,32]. However, the sheer diversity of pathogens coupled with their ability to use virulent resource-acquisition strategies means exploitative competition from commensals is unlikely to provide perfect pathogen resistance.

*Interference competition* refers to direct negative interactions within and between species via behaviours that interfere with the others’ ability to survive and grow. Microbes engage in both active and passive forms of interference. Microbes commonly carry weapons to kill their competitors, such as systems to secrete toxins into the environment or directly into opponent cells and carrying prophage that can trigger viral epidemics [11,33]. Other methods of active interference include secreting molecules that disrupt competitors’ biofilms or cell-to-cell communication [34,35]. These mechanisms are frequently found employed by commensals against pathogens [13,34,35], but notably are also in wide use by pathogens [11]. More passive interference can arise as a side-effect of a microbe’s resource acquisition strategy. For example, commensal *Lactobacillus* species in the human vagina produce lactic acid as a by-product of glycogen metabolism; the resulting acidity inhibits non-*Lactobacillus* species, including several pathogens [36,37]. Biofilm formation also interferes with the ability for other microbes to access nutrients. In the mammalian large intestine, for example, commensals biofilms in the outer mucus layer deny competitors access to nutrients in the inner mucus layer and physically separate pathogens from the vulnerable host tissues beneath [13].

Interference can alter the outcome of competition by allowing a competitor to overcome a relative disadvantage in resource exploitation ability [15,38]. The harm received from interference mechanisms typically increases with the population density of the harming species [8,38], putting resident commensals at an inherent advantage over rare pathogenic invaders. But spatial structure enhances the effectiveness of interference from rare invaders, and self-replicating phage-based weapons can be effective when used by a rare invader even in a well-mixed environment [8]. Disruption of the microbiome (e.g. due to antibiotic treatment) can temporarily abate commensal interference and resource consumption, allowing an otherwise resisted pathogen to invade and subsequently use its own interference mechanisms to prevent the return of the commensal community. Such dynamics are important drivers of the human microbiome-related diseases of *C. difficile* infection [39] and bacterial vaginosis [40].

Macroecologists recognized that natural enemies such as predators and parasites create an additional pathway for negative feedbacks between species. *Apparent competition* [41,42] arises when the increase in a focal species drives an increase in the enemy, which in turn causes increased direct harm to that species and any others that share the enemy. Perhaps the most natural analogy to the microbial world would place bacteriophage in the role of the enemy, and indeed phage are increasingly recognized as an important regulator of human microbiomes through this mechanism [10,43–45]. But the natural enemy of host-associated microbes most pertinent for pathogen resistance is probably the host immune system [46,47].

The species that triggers an immune response may ultimately benefit, if the direct harm it receives is outweighed by the indirect benefit of killing off its competitors. The standard model of immune defence is that a pathogen activates an immune response that kills said pathogen. But a host immune response to one microbe can harm all microbes in the affected tissue region and even cause a systemic immune response [35,47–49]. Several mammalian pathogens suppress commensal competition by triggering immune responses that they are more resistant to than their competitors [8,50,51]. The reverse has also been observed in a plant system and an animal system, where the pathogen resistance apparently conferred by a commensal was found to be at least partly driven by the commensal’s triggering a host immune response that primarily harmed that pathogen [48,52]. It also appears to be common among plants and animals for the presence of commensals to ‘prime’ the immune system into an alert state that responds more rapidly and severely when a pathogen is encountered [35,49]. Commensals can also prompt changes in physical barriers, such as increased mucus production in the mammalian large intestines [53] or increased leaf toughness in plants [48], that penalize microbes that seek to violate host tissues more than those content to inhabit their outer surfaces.

## 3. Natural selection favours hosts that cooperate with commensals to resist pathogens

To infect a host, the pathogen must overcome both the host’s innate defences and competition from commensals. Thus pathogen resistance is ultimately a joint property of the host and its microbiome. We argue that natural selection favours hosts that cooperate with commensals to resist pathogens by directly facilitating commensal growth and by limiting the collateral damage commensals receive from the host’s own anti-pathogen defences.

Innate host defences are unable to resist all relevant pathogens. Hosts use physical barriers and a variety of more sophisticated means for killing microbes; however, pathogens are diverse and evolving targets and most defence mechanisms also carry a heavy cost to the host. Mechanisms to contain, kill or expel microbes trade off with the host’s needs for self-maintenance, growth and reproduction. Each individual defence mechanism will also face diminishing returns in the fraction of additional pathogens resisted per unit cost because the pathogens that are not yet resisted will be enriched in behaviours or phenotypes that inherently withstand the given defence. Hosts can to some extent overcome diminishing returns in one mechanism by using multiple defences. But it is apparent that in practice the optimal level of defence is both extremely costly and far below perfect resistance [4–7,54]. Thus innovations that increase defence while escaping existing trade-offs can provide substantial net benefits, even if they bear their own substantial costs.

One such innovation is to leverage the competition against pathogens from the commensal microbiome. Commensals are expected to decrease the initial growth rate of an invading pathogen through exploitation and interference competition, independent of the host. As a result, the mere presence of commensals can decrease the fraction of pathogens that are able to successfully infect the host. But commensals are also at the mercy of the host’s defences. A host defence that kills commensals indirectly benefits pathogens by removing their competitors. This is true for constitutive host defences (such as constitutive production of antimicrobial peptides) and facultative host defences (such as an inflammatory response in animals or induced systemic immunity in plants)—even if the facultative defence is triggered by the pathogen. Thus, pathogen growth rate is minimized by innate defences that kill pathogens while leaving commensals unharmed. Host mechanisms that facilitate commensal growth can further decrease pathogen growth rates as those commensals provide increased interference or are able to further consume resources particularly limiting to pathogens.

**Figure 2.**
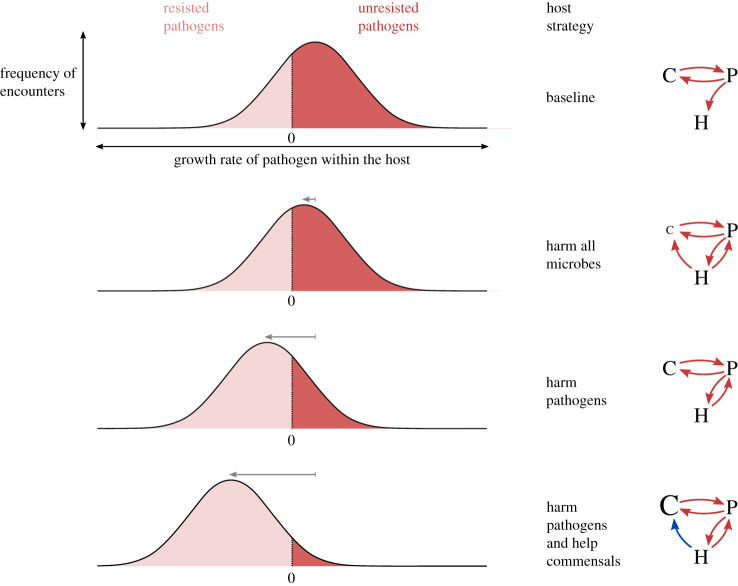
A conceptual model of selection for cooperative immunity. Hosts encounter random pathogens with varied innate infectivities, which we represent by a distribution of exponential growth rate across pathogens. Encountered pathogens with positive growth rates successfully infect the host and those with negative growth rates are resisted. Host fitness increases when the fraction of unresisted pathogen encounters (dark red area under the curve) is reduced. Each row illustrates a different host defence strategy, illustrated by the arrow diagrams, in which red arrows denote direct harm and blue arrows denote direct help between host (H), commensal (C) and pathogen (P). Grey arrows indicate the shift in the peak of the distribution of pathogen growth rates from baseline caused by the given strategy. Row 1: As a baseline, we consider a host that is colonized by commensals that compete with its pathogens. Row 2: A host strategy that harms commensals and pathogens equally reduces the competition experienced by pathogens and so has a limited net impact on pathogen growth rates. Row 3: Selectively harming pathogens maintains competition from commensals and so provides a greater reduction in pathogen growth rates than harming all microbes. Row 4: Additionally helping commensals increases the competition from commensals and thus further decreases pathogen growth rates. The host may also have a fixed set of baseline antimicrobial defences common to all strategies, which we do not represent in the arrow diagrams.

Whether and how hosts ultimately use commensals is the outcome of population-level selection on host genotypes. Figure 2 describes a conceptual model for how pathogen pressure might select for hosts to tolerate and even encourage colonization by commensals. We consider a host that encounters pathogens throughout its lifetime and consider the fitness of the host to be inversely proportional to the expected fraction of these encounters that successfully lead to an infection (the dark red area under the curves in figure 2). For simplicity, we suppose the ability for pathogens to infect the host and the ability for the host and its microbiome to resist them can be quantified by one-dimensional numbers. The outcome of invasion by a pathogen is then determined by whether its intrinsic ability to establish within the host environment is greater than the combined resistance of the microbiome and innate host defences.

We take as our starting point a host with a baseline level of innate defences that harbours a commensal microbiome capable of resisting a non-negligible fraction of pathogens that the host encounters (figure 2, row 1). We consider this a reasonable default given the impracticality of completely eliminating commensals and the likelihood that any microbiome provides at least some resistance. In this context, a new host trait that harms all microbes can improve pathogen resistance, but not as much as would be naively expected because such a trait will also reduce the competitive resistance provided by commensals (figure 2, row 2). A host trait that can be targeted to harm pathogens and not commensals will offer a great improvement to pathogen defence since the commensal competition is maintained (figure 2, row 3). Finally, a host trait that can be targeted to increase commensal colonization or select for commensals particularly good at excluding pathogens can also improve pathogen resistance, even in the absence of any direct suppression of pathogens by the host (figure 2, row 4). This simple model makes clear how host defence traits that modulate the commensal microbiome can translate into fewer infections and thus increased host fitness, allowing them to spread through the population by natural selection.

Drawing on others who remarked on cooperative relationship between commensals and host immune systems [13,35], we use the term *cooperative immunity* to refer to this evolutionary strategy in which the host helps commensals in order to increase its own pathogen resistance. Importantly, the above argument for the evolution of cooperative immunity does not require any ongoing evolution (or co-evolution) of the commensal microbes with the host. Resistance provided by commensals arises from adaptations in those lineages for competing with other microbes, not for the benefit of their host.

Whether cooperative immunity is expected to evolve ultimately depends on at least two additional factors. First, there must be heritable variation in host traits that regulate competition between commensals and pathogens for selection to act upon. We discuss evidence and examples of such traits next. Second, the benefit to resistance must exceed the costs of employing a cooperative immunity strategy. Beyond the energetic and developmental costs of any specific mechanism, encouraging or tolerating commensals can cost the host nutrients [55] and can leave the host more vulnerable prior to developing a fully formed microbiome or following a stress that disturbs the microbiome. However, we argue these drawbacks will often be more than overcome by the potential fitness benefit of even a small increase in resistance given the significant impact microbial pathogens have on most hosts.

## 4. Host traits can influence microbial competition in favour of commensals over pathogens

Microbial competition within a host plays out in an environment over which the host has much control, and that it can modify in ways that could influence microbial interactions towards favourable (to it) competitive outcomes.

### (a) Regulation of available resources

To maximize pathogen resistance, it might seem that hosts should minimize the nutrients that support microbial growth in their immediate exterior. In fact they seem to do the opposite. Plants secrete 5–25% of their photosynthetically fixed carbon into the surrounding soil as mucilage, where it is consumed by microbes [56–58]. Humans feed the great mass of bacteria in their large intestine with non-host-digestible polysaccharides as well as carbohydrates secreted by host epithelial cells into intestinal mucus [53,59]. Mammal and bird skin contains glands that harbour commensal microbes and secrete lipids they can consume [60,61].

Microbial provisioning by hosts has several non-mutually exclusive explanations. Plant mucilage can lubricate roots and stabilize soil [56], and animal consumption of non-digestible polysaccharides can be a side-effect of eating plants for their digestible components. Mucilage and mucus secretions can also serve as a direct antimicrobial defence; both contain antimicrobial peptides and can increase in the presence of pathogens [56,62]. But it is clear that plant root secretions, and dietary and secreted nutrients of mammals, substantially increase the total microbial density in the rhizosphere and gut and can significantly influence its species composition relative to the environment [56,57,63,64]. Hosts obtain large direct benefits from these microbes in the form of nitrogen fixation from bacteria and mineral uptake from fungi in plants [65] and the conversion of plant polysaccharides into host-digestible nutrients in animals [53,59,66]. We argue that an additional function of microbial provisioning in these and other tissues is to encourage a commensal microbiome that can successfully outcompete pathogenic invaders.

The general provisioning of energy resources would seem to encourage the growth of commensals and pathogens alike. However, hosts can obtain a favourable outcome if commensals get first access to these resources, as then the commensals can establish a dense population that subsequently inhibits pathogens by physically occupying host surfaces, engaging in interference, and consuming other limiting nutrients as described above. In many hosts, physical contact or proximity can help to spread commensals quickly from already colonized hosts to newly provisioned host environments. In animals, specialized behaviours [67–69] can ensure that provisioned surfaces and compartments are immediately colonized by commensals early in life. In addition, nutrient secretions may favour non-pathogenic microbes if the ability to exploit nutrients external to host tissues trades off with pathogenic phenotypes for invading or disrupting host tissues.

To some extent, hosts can also direct nutrients specifically to commensal species. For example, chemical modifications of secreted carbohydrates in roots and intestinal epithelium can also allow hosts to partially control which species are able to consume them [53,56,58]. One example is the oligosaccharides secreted in human breast milk that support the growth of *Bifidobacteria* [70]. Hosts can also locally adjust resources in response to detecting specific microbes or microbial activity, providing nutrients in response to commensals and restricting them in response to pathogens [28,62].

### (b) Preferentially harming pathogens

Hosts directly harm microbes by producing antimicrobial peptides or toxins and (in animals) destroying microbes with specialized immune cells. A variety of factors influence which microbes are affected by these antimicrobial defences, which hosts use to preferentially harm pathogens. By targeting defences at pathogens, hosts limit costly immune responses and avoid killing microbes that provide nutrients or other direct benefits. Yet a perhaps greater benefit is to maintain and enhance the competitive resistance provided by commensals. Killing microbes indiscriminately would decrease the density of the commensal microbiome and thus the degree of inhibition exerted on invaders (figure 2). Harming only pathogens maintains and adds to the inhibition from commensals to broaden the range of resisted pathogens. Two general host strategies for targeting antimicrobial activity are to distinguish between pathogenic and commensal species and to distinguish between the damaging and non-damaging behaviours exhibited by said species [58].

Hosts have some ability to direct their harm by distinguishing between commensal and pathogen species. The antimicrobial peptides (AMPs) produced by plant and animal hosts vary in their effects on microbial species. The distinct constitutive AMP profiles of closely related *Hydra* species were found to be capable of selecting for distinct microbiotas [71]. All hosts have the ability to detect microbial cells by using pattern-recognition receptors (PRRs) to recognize specific molecules on microbes’ surfaces, known as microbial-associated molecular patterns (MAMPs), which trigger an innate immune response. Some plant PRRs recognize specific secretion systems that are more prevalent among pathogens [72]. Most PRRs recognize molecules that evolve slowly and are found in commensals and pathogens, but even these PRRs can have some species specificity; for example, the flagellin of the grapevine endophyte *Burkholderia phytofirmans* is recognized differently from that of two grapevine pathogens [72].

A more robust and universal strategy is to punish *pathogenic behaviour* rather than pathogen species [58]. In the gut, AMPs and immune receptors are concentrated near the epithelial layer; commensal microbes are shielded by the mucus layer, and pathogens that degrade or invade it in their attempt to breach the epithelium are exposed [73,74]. Recognition of damage can trigger local inflammation, so that commensals away from the immediate infection site will be unharmed [58]. Similarly, epithelial cells in the chicken gut express an extracellular receptor that triggers a local inflammatory response when destroyed; it is protected from microbial proteases until the mucous layer is breached by pathogenic yeast or bacteria [54, p. 47]. Plants store toxins in specialized tissue structures that are released only when microbes or herbivores destroy the plant’s tissue and so will not affect commensals in undamaged sites [28, ch. 18]. Plant and animal immune systems avoid attacking just any microbe they encounter by supplementing microbial pattern recognition with damage sensing. In particular, hosts have the ability to detect damage via PRRs that bind molecules such as extracellular DNA and ATP that are only released extracellularly when their own cells are disrupted [75]. Requiring detection of such damage-associated molecular patterns (DAMPs) in addition to MAMPs can help ensure that pathogens are disproportionately killed by host immune responses.

## 5. Broader implications of cooperative immunity for host evolution

Beyond the evolution of host traits to foster and maintain a pathogen-resistant microbiome, the incorporation of commensals into a host’s pathogen defence strategy can have broader implications for its long-term evolution.

### (a) New evolutionary opportunities from more effective and efficient pathogen defence

If the incorporation of commensals into a cooperative immunity strategy provides stronger or less costly pathogen defence to the host, it can serve to unlock areas of host phenotypic space that otherwise would be selectively inaccessible owing to increased pathogen risk. As our motivating example, we consider epithelial tissues—the barriers between higher organisms and the environment. Epithelial tissues in animals, including the skin, intestinal and respiratory epithelia, are where the crucial exchanges of nutrients, oxygen and waste occur between the host and the external environment. Each of these epithelial tissues also hosts significant commensal microbiota in most animal species. The same pattern is seen in plant roots, which are responsible for absorption of the majority of nutrients and water for the whole plant and thus are in close contact with a microbially dense environment and are highly associated with a microbiome of commensals and symbionts [76]. We suggest that the incorporation of the commensal microbiota into the critical function of pathogen defence has made these epithelia on the whole more efficient and less costly for animals and plants to maintain.

Gains in efficiency and lower costs for critical tissues such as epithelia allow the evolutionary process to explore new areas of phenotype space. First, if policing epithelial pathogen exposure is less costly, then it can be evolutionarily feasible to develop larger epithelia, or at least epithelia that function at a higher rate of exposure to the external environment, such as a mussel that filters 10 litres of water per hour instead of five. Second, resource savings relative to a no-microbiome pathogen defence strategy can be reinvested in other areas of growth and development. As an analogy, the ‘cooking hypothesis’ proposes that the energetic and time savings gained by a transition towards eating cooked meat unlocked the resources needed for modern humans to develop our large brains and, thereby, complex behaviours and societies [77]. We suggest that, in similar fashion, as multicellular hosts adapted to and co-opted their microbiome into a more efficient cooperative immunity against the pathogen menace, benefits were realized by the evolutionary process in other unrelated phenotypic areas.

### (b) Initiation of strong associations and long-term co-evolution between hosts and specific microbes

There are many striking examples of commensals that provide direct benefits to their hosts, such as the ability to digest wood in termites [78], fix nitrogen in plant roots [79] and use bioluminescence for active camouflage in the bobtail squid [80]. In these cases, opportunity for mutualism occurred because a preexisting selfish ability of the symbiont matched a preexisting need of the host; e.g. a microbe that derives nutrition from wood converts it into something more digestible to the termite. But the intricate physiological interdependencies that formed required long-term stable co-evolutionary associations between host and symbiont, which during their initiation and throughout required that the costs of tolerance of and investment in each partner were outweighed by the benefits gained. We suggest that in many cases the pathogen resistance provided by these microbes was an important term in the cost–benefit calculation leading to their stable role as providers of non-pathogen-mediated benefits, especially early on in their co-evolutionary history.

The ubiquity of microbial competition means that any commensal with a potential direct benefit will compete against at least some of the pathogens that afflict a host. Thus, we expect such microbes to provide some pathogen resistance more often than not, something that has in fact been observed in many direct mutualisms. For example, the same microbes in the human gut that convert dietary fibre into short chain fatty acids usable by the host also provide pathogen resistance through the various types of microbial competition we described above [81]. Thus, pathogen resistance can be a supplemental or even the primary benefit initially provided by the commensal. Importantly, benefits to pathogen defence require no prior evolutionary history between the host and the microbe, and are realized even when commensals are only loosely associated and intermittently present (albeit reduced proportional to the fraction of time the host is colonized by commensals). Facilitation of commensal colonization by the host that initially evolved for the purposes of resistance may increase the degree of non-pathogen-related benefits received by the host and begin stronger host–microbe associations. Subsequent co-evolution can enhance the direct benefit provided by the symbiont to the point seen in the examples opening this section, where the host’s way of life depends on it. Furthermore, as hosts evolve to manage commensal microbes that provide them pathogen defence, the mechanisms they develop to regulate the microbiota also serve to regulate those that provide direct benefits.

## 6. Pathogen resistance: the principal evolutionary advantage of the microbiome?

To be in consideration for the principal evolutionary advantage, a microbiome-derived function should be widespread across many hosts and offer clear evolutionary benefits to the host. Many microbiome-derived functions involve a highly specific match between microbial ability and host need—for example, luminescence provided by *Vibrio* species to the bobtail squid [80], or insecticide resistance provided to related stinkbug species by fenitrothion-degrading strains of *Burkholderia* [82]—but do not apply to a broad range of hosts. The need in many animals for microbial signals for normal development and homeostasis may be a side-effect of evolving in a context where such signals are typically present, even if using those signals provides no short- or long-term advantage [49,63]. Of non-pathogen-related benefits, those to nutrition best satisfy the two conditions we propose for consideration as the principal evolutionary advantage of the microbiome. All hosts need nutrients to survive and grow, and the diversity of microbial metabolism provides myriad ways for microbes to contribute to fitness-limiting aspects of host nutrient acquisition. Many insects and mammals use gut microbes to convert plant matter into digestible and/or non-toxic forms and to synthesize essential nutrients [63]; most plants use mycorrhizal fungi to gather minerals and/or root-associated bacteria to fix nitrogen [83–85].

We believe that pathogen defence also merits consideration as the principal evolutionary advantage of the microbiome. Perhaps the strongest argument in favour of pathogen resistance is the apparently ubiquitous opportunity for hosts to gain a substantial pathogen-resistance fitness benefit simply by being colonized by commensals. Death or injury from pathogen infection is a universal fact for plants and animals, and the strong selection to resist pathogens has driven the evolution of a multitude of innate defence mechanisms across the tree of life. However, all innate defence mechanisms are constrained by trade-offs with other host needs. Thus, there is a strong potential benefit for hosts that can also leverage extrinsic factors to increase pathogen resistance. Commensal microbes are just such a factor. Commensals are ever-present, and naturally provide pathogen resistance through the innate competition between microbes over resources and via the many mechanisms of microbial warfare. There is no taxonomic or functional restriction on the microbes that can provide pathogen resistance beyond the ability to harmlessly persist in the host, and the mechanisms by which commensals can suppress pathogens require little if any prior co-evolution with the host. Thus many commensals are likely capable of suppressing multiple pathogens, and a diverse commensal microbiome is likely capable of suppressing a wider variety of pathogens still. Compared with nutritive benefits, microbiome benefits to pathogen resistance are more diffuse (spread across a range of possible pathogen encounters) and redundant (could be provided by many microbes that might have colonized the host). Thus, while it is undeniable that the benefits granted to the host by many specific commensals are dominated by non-pathogen functions, pathogen resistance may still dominate the benefit of the microbiome as a whole. And pathogen defence benefits might be more robust in the loosely associated microbiomes of highly variable taxonomic compositions that are observed in many hosts, and that likely predominated at the critical early stages of the evolution of close symbioses between hosts and some highly adapted symbionts.

The ability for commensals to provide resistance against pathogens has long been known, and interest in leveraging the microbiome to improve plant and animal health is increasing. Yet the pathogen-resistance benefit of the microbiome is still often missing from discussions of the impact of the microbiome on host ecology and evolution. Here we consider the question of whether pathogen resistance may be the most important microbiome function in the broadest evolutionary terms. We do not downplay the obvious importance of the many other functions that the microbiome provides, but are purposefully highlighting the vast and potentially underappreciated impact of commensal-derived pathogen resistance throughout the evolution of plants and animals. We hope that increased attention may help advance our understanding of the evolution of host–microbe interactions, and the nature of immunity, and improve microbiome-based diagnostics and therapeutics in the future.

## Data accessibility

This article has no additional data.
